# Deciphering the potential roles of ferroptosis in regulating tumor immunity and tumor immunotherapy

**DOI:** 10.3389/fimmu.2023.1137107

**Published:** 2023-02-28

**Authors:** Xu Gu, Yu’e Liu, Xiangpeng Dai, Yong-Guang Yang, Xiaoling Zhang

**Affiliations:** ^1^ Key Laboratory of Organ Regeneration and Transplantation of Ministry of Education, First Hospital of Jilin University, Changchun, China; ^2^ National-Local Joint Engineering Laboratory of Animal Models for Human Disease, First Hospital of Jilin University, Changchun, China; ^3^ Tongji University Cancer Center, Shanghai Tenth People’s Hospital of Tongji University, School of Medicine, Tongji University, Shanghai, China; ^4^ International Center of Future Science, Jilin University, Changchun, China

**Keywords:** programmed cell death, ferroptosis, cancer, immunotherapy, tumor microenvironment, resistance, immune checkpoint inhibitor

## Abstract

Cancer immunotherapies, including immune checkpoint inhibition (ICI) and adoptive immune cells therapy, are promising therapeutic strategies. They reactivate the function of immune cells and induce immune responses to attack tumor cells. Although these novel therapies benefited a large amount of cancer patients, many cancer patients have shown fair responses even resistance to cancer immunotherapies, limiting their wide clinical application. Therefore, it is urgent to explore the underlying mechanisms of low response and resistance of cancer immunotherapy to enhance their treatment efficacy. The programmed cell death (PCD) including the ferroptosis, has been demonstrated to play essential roles in antitumor immunity and in regulating the immune response to ICIs. Ferroptosis, a phospholipid peroxidation-mediated, iron-dependent membrane damage, exhibite three critical hallmarks: the oxidation of phospholipids, the lack of lipid peroxide repair capability and the overloading of redox-active iron. Notably, ferroptosis was found to plays important roles in regulating tumor immunity and response to immunotherapy. Therefore, targeting ferroptosis alone or in combination with immunotherapy may provide novel options to promote their antitumor efficacy. However, the effect of ferroptosis on tumor immunity and immunotherapy is affected by the interaction of ferroptosis and cancer cells, immune cells, tumor microenvironment (TME) and others. In this review, we summarized and discussed the critical roles of ferroptosis in regulating antitumor immunity, TME and in the improvement of the therapeutic efficacy of immunotherapy in cancers.

## 1 Introduction

**Figure 1:**
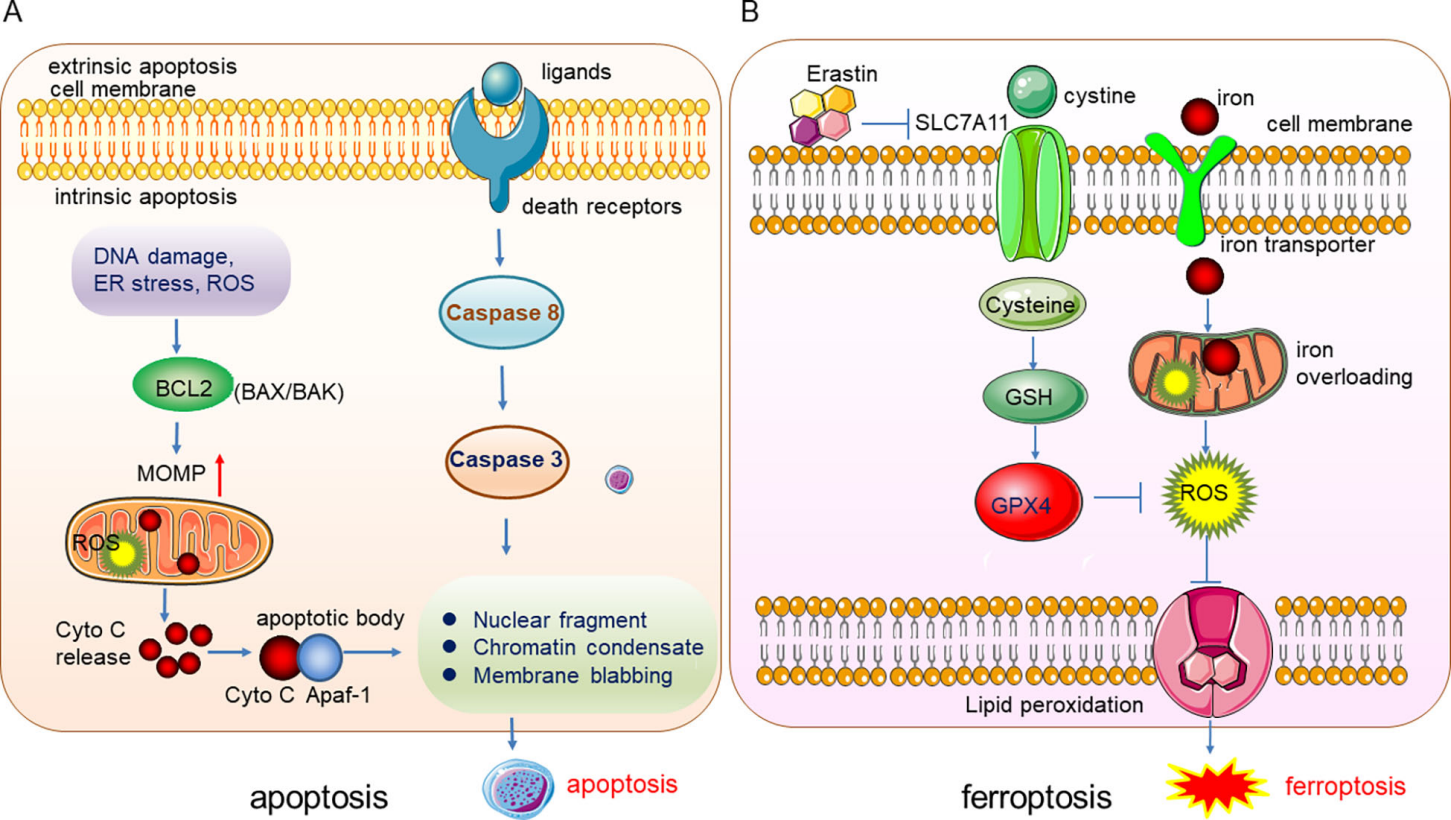
Illustration of the underlying mechanisms of apoptosis and ferroptosis. **(A)** The phenotype of apoptosis is characterized by the nuclear fragment, chromatin condenses and cell membrane blabbing. **(B)** Ferroptosis is mainly regulated by iron homeostasis and oxidative stress.

Ferroptosis, a form of programmed cell death (PCD), was first found and termed by Dixon in 2012. It is a lipid peroxidation-mediated, iron-dependent membrane damage different from other PCDs such as apoptosis, necrosis, and autophagy in morphological, biochemical, and genetic characteristics (1). The specific difference between ferroptosis and apoptosis lies in the difference in the morphology and the organelles, and the biomarkers. The apoptosis can be induced by the extrinsic (ligand-receptor interaction) and intrinsic (mitochondria) pathways, the death ligands and receptors interaction recruits Capase 8 and triggers the activation of Caspase 3 which degrades the downstream functional proteins and lead to apoptosis. The intrinsic apoptosis can be induced by DNA damage, reactive oxygen species (ROS), endoplasmic reticulum (ER) stress, etc. These stresses activate the pro-apoptis proteins in the BCL2 family such as BAX/BAK and thus leading to the depolerlization of mitochondria out membrane potential (MOMP), and the production of ROS, subsequently, cytochrome C (Cyto C) were released from the mitochondria and binds to Apaf-1 to form the apoptotic body to induce the apopotis. The phenotype of apoptosis is characterized by the nuclear fragment, chromatin condenses and cell membrane blabbing (**Figure 1A**). While in ferroptosis, there is no chromatin condensation or loss of plasma membrane integrity, but mitochondria condensation and the mitochondria membrane potential increases (2). Ferroptosis is mainly regulated by iron homeostasis and oxidative stress (**Figure 1B**). Iron overloading and ROS accumulation lead to lipid phosphorylation of the cell membrane and trigger ferroptosis. Cysteine was transported into the cell by transporter SLC7A11 and synthesized the glutathione (GSH), which promotes the activity of the important anti-oxidant enzyme glutathione peroxidase 4 (GPX4), GPX4 reduces the quantity of ROS and maintain the redox homeostasis. Once the GPX4 activity is inhibited, the accumulation of ROS will lead to lipid peroxidation and ferroptosis will be triggered subsequently (**Figure 1B**).

Erastin, a RAS-selective lethal small molecule, could induce cancer cell ferroptosis by inhibiting the activity of SLC7A11. Triggering ferroptosis by small molecules or other methods could benefit the killing of some tumor cells (1). Given that ferroptosis is a specific cell death caused by iron-dependent lipid peroxidation, three critical hallmarks of ferroptosis have been specified: the oxidation of phospholipids, the lack of lipid peroxide repair capability and the availability of redox-active iron (3). Therefore, some biological processes or cell singling pathways associated with the three essential hallmarks might govern the ferroptosis function. For example, it was reported that GPX4, ferroptosis suppressor protein 1 (FSP1), amino acid, iron metabolism and lipid peroxidation play important roles in regulating ferroptosis function (4). The FS1-CoQ10 pathway, GCH1-BH4 pathway, and the DHODH pathway also suppress ferroptosis (5).

It is well-known that the immune surveillance of the immune system plays critical roles in monitoring, identifying, killing and eliminating tumor cells at different stages. However, immune surveillance can be bypassed or escaped by tumor cells through decreasing the immunogenicity and inducing an immunosuppressive status (6). Therefore, reactivating the functions of the immune cells and inducing immune responses to tumor cells are promising therapeutic strategies. These advanced methods include chimeric antigen receptor T (CAR-T) cells (7), immune checkpoint inhibition (ICI) (8), cytokine therapies (9) and dendritic cell vaccines (10). ICI, a novel anti-tumor therapeutic strategy, has made huge progress in treating multiple cancer malignancies efficiently in recent years. It reactivates tumor monitoring functions of effector T cells by blocking immune checkpoints such as programmed cell death-ligand 1 (PD-L1) and cytotoxic T lymphocyte-associated antigen-4 (CTLA-4) and subsequently causes tumor cell death. However, a large number of tumors have shown fair response even no response to ICI treatment, probably due to the low level or deficiency of tumor-infiltrating lymphocytes (TILs), which limited the wide clinical application of ICI in tumors. Therefore, it is urgent o explore the underlying mechanisms for the resistance to tumor immunotherapy and dig for novel methods (11). Interestingly, among those investigated mechanisms, the programmed cell deaths (PCDs) attracted many attentions and they were found to play essential roles in antitumor immunity and in regulating the immune response to ICIs.

Although the perforin-granzyme and the Fas-Fas ligand (FASL) signaling pathway were considered the canonical mechanisms for the function of immunotherapy-induced CD8^+^ T cells in inducing cancer cell death (12, 13), it was also reported that the CD8^+^ T cells can inhibit tumor cells through the induction of ferroptosis and pyroptosis (14, 15).

Moreover, targeted therapies for ferroptosis inhibition or induction alone or in combination with immunotherapy may provide novel options to promote the antitumor efficacy of immunotherapeutic strategies. Interestingly, different types of tumors showed diverse sensitivity to the treatment strategy targeting ferroptosis. It was reported that the highly aggressive triple-negative breast cancer (TNBC) is sensitive to a limited number of targeted therapies but showed high vulnerability to the treatment of ferroptosis induction. Furthermore, ferroptosis was also reported to play critical roles in anti-tumor immunity by regulating T cell function and in modulating the efficacy of tumor immunotherapy (16). Therefore, ferroptosis attracts increasing interest in understanding its potential roles in cancer therapy due to the specific tumor suppressive signaling pathways and the feasibility of the development of ferroptosis-inducing drugs (17, 18). In this review, we summarized and discussed the potential roles of ferroptosis in regulating antitumor immunity, tumor microenvironment (TME) and the improvement of therapeutic efficacy of immunotherapy in cancers.

## 2 Ferroptosis and tumorigenesis

It is well-known that human body removes and cleans the unneeded, abnormal or damaged cells through PCD such as apoptosis to ensure the normal development of organs and maintain homeostasis. Therefore, various diseases including cancers will be induced by the dysfunction of PCD. Moreover, cancer cells could be subjected to uncontrolled growth by blocking the PCD. In recent years, as a novel form of PCD, ferroptosis attracted enormous attention for its function in tumorigenesis and induction of ferroptosis might be a novel attractive therapeutic strategy for cancer treatment.

**Figure 2:**
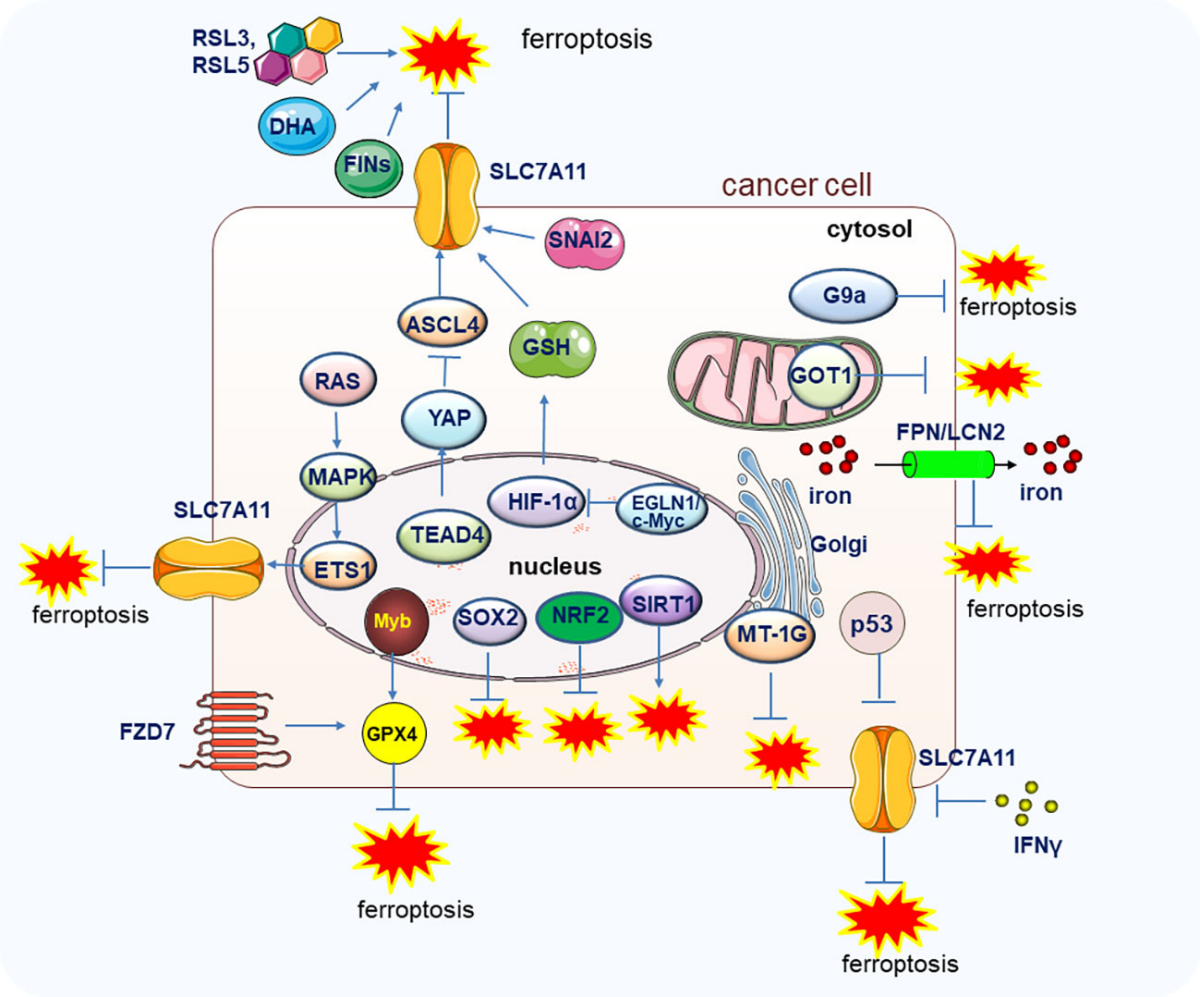
The genes and pathways involved in the regulation of ferroptosis in tumor cells. Main genes and signal pathways involved in the regulation of ferroptosis in tumors cells are illustrated.

Like other forms of PCD, ferroptosis may also play a crucial role in suppressing cancer development. As early as 2003, Erastin was found to trigger cell death in engineered cells by inducing ferroptosis (19). Moreover, in 2012, the Cystine/glutamate antiporter xCT (SLC7A11), which modulates the intracellular glutathione levels through regulating cystine-glutamate exchange, was found to exert resistant function on ferroptosis (1), SLC7A11 has been found to be overexpressed in various cancers to rescue the tumor cells from ferroptosis mediated cell death (20, 21). Notably, the oxidant and the antioxidant pathways contribute to the transformation of the proto-oncogene *RAS* which is highly mutated and abnormally activated in many kinds of cancers, but the underlying mechanisms are not fully investigated. A recent study uncovered the function of SLC7A11 on RAS-induced transformation and tumorigenicity by increasing antioxidant glutathione synthesis. Mechanically, the transcription factor ETS-1 as the downstream of the MAPK signaling pathway synergized with ATF4 to directly activate SLC7A11, thus, the tumor growth in KRAS-transformed cells xenograft was inhibited upon depletion of SLC7A11 (22). In line with this finding, in cisplatin-resistant head and neck cancer (HNC) cells, inhibition of SLC7A11 could recover their sensitivity to cisplatin treatment *in vitro* and *in vivo* (23). It is well known that the famous tumor suppressor p53 could inhibit tumorigenesis by a canonical mechanism including inducing cell cycle arrest, senescence, and apoptosis. However, researchers also found that the p53 could retard tumor growth through a non-canonical mechanism by inducing ferroptosis *via* suppressing the SLC7A11 expression. The acetylation-defective mutant p53^3KR^ losses the capability to induce apoptosis, cell cycle arrest and senescence but still preserves the ability to induce ferroptosis by reducing the expression of SLC7A11 under the ROS-mediated stress (24). *SNAI2* showed oncogenic function to promote ovarian cancer progression by upregulating the SLC7A11 to inhibit ferroptosis (25). Importantly, the expression level of GPX4, the most important regulator of ferroptosis, could markedly affect the renal cell carcinoma cell growth by regulating ferroptosis, and the ferroptosis could be reversed by the treatment of vitamin E and iron chelator desferrioxamine (DFO) (26, 27). Furthermore, the EGLN1/c-Myc/HIF-1α/lymphoid-specific helicase (LSH) signaling axis could promote the tumor cell growth associated with WDR76 by blocking ferroptosis through the activation of lipid metabolism-related genes (28). It was also reported that the proto-oncogene Myb could positively regulate cell proliferation by inhibiting ferroptosis *via* the cysteine dioxygenase 1 (CDO1)–GPX4 signaling axis (29). Moreover, the TEA domain transcription factor 4 (TEAD4) exerts its oncogenic function by negatively regulating ferroptosis through the activation of Yes-associated protein (YAP) and subsequently affecting the expression of TFRC and ACSL4 (30). Therefore, the exploration of more regulation mechnasms of ferroptosis and in deep investigation of the specific molecules or pathways in regulating ferroptosis will shed light on the manipulation of ferroptosis to combat tumors (**Figure 2**).

Altogether, these findings suggest that the induction of ferroptosis by small molecules or other methods directly targeting ferroptosis or targeting the related pathways could be a novel therapeutic strategy for cancer treatment.

## 3 Ferroptosis and tumor microenvironment

Both the tumor-related genes and the TME influence the function of ferroptosis in tumorigenesis, tumor immunity, and tumor therapy. The TME is a multicellular environment which include extracellular matrix, immune cells, blood vessels, tumor cells and other cells. TME plays critical roles in regulating tumor occurrence, progress, metastasis and drug resistance through influencing metabolic, epigenetic, immune or other components in the microenvironments (31). For example, ROS, one of the important inducing factors, can be generated by heat and radiation exposure, metabolism, redox homeostasis, and other environmental and genetic stimuli, all of which are important components of TME (32). Furthermore, it was reported that the oxidized lipid mediators, eicosanoids, ATP, HMGB1 and other signal molecules could be released into the TME by ferroptotic cancer cells (33–35). The iron-carrier protein transferrin and extracellular amino acid glutamine in the TME were found to have the capability to induce ferroptotic cell death (36). Furthermore, the effector CD8^+^ T cells could produce IFNγ to reduce the cancer-associated fibroblasts (CAFs) released cysteine and GSH and subsequently decreased the expression of *SLC7A11* and *SLC3A2*. The cystine uptake was inhibited in cancer cells with low expression of *SLC7A11* and *SLC3A2* which ultimately triggered the tumor lipid peroxidation and ferroptosis (37). Therefore, these findings demonstrated that the TME and ferroptosis could affect each other to regulating tumor development and immunity.

### 3.1 The interaction between ferroptosis and immune cells in TME

Given that some immune cells including T cells, macrophages, myeloid-derived suppressor cells (MDSCs), DCs, B cells, and natural killer (NK) cells are important components of TME and highly correlated with ferroptosis, a better understanding of their crosstalk with ferroptosis will help to provide a new option for the regulation of cancer immunity and the development of ferroptosis-targeted therapy (38).

#### 3.1.1 Ferroptosis regulates the function of T cells and Tregs

Notably, lipid accumulation is a common metabolic alteration in TME and is highly associated with immune abnormality. It was reported that the lethal lipid peroxides (LPOs) quickly accumulated in the membrane of T cells depleted of *GPX4* which will subsequently undergo ferroptosis and lack the capability to expand and protect the cells from infection *in vivo* (39). Consistently, ferroptosis might be an important metabolic regulator of tumor-specific CD8^+^ T cells. It was found that the activated CD8^+^ T cells and CD4^+^ T cells were more sensitive to GPX4 inhibitors than antigen-expressing cancer cells in an *in vitro* co-culture system (40). It was found the GPX4 inhibitors could decrease the killing efficiency of CD8^+^ T cells at a concentration which did not affect the growth of cancer cells. The result suggested that the low dose of GPX4 inhibitors will benefit the growth of cancer cells by selectively inhibiting the killing function of CD8^+^ T cells. Interestingly, the reduced killing efficacy of T cells could be rescued by ferroptosis inhibitor ferrostatin-1. Furthermore, they found that the acyl-CoA synthetase long-chain family member 4 (ACSL4) mediated the sensitivity alteration and ferroptosis induced by GPX4 inhibitors in activated CD8+ T cells (40). However, there might be some conflicting conclusions in studies that used different methods to induce ferroptosis. The sensitivity to pro-ferroptotic stimulation of tumor cells and CD8+ T cells could also affect the function of ferroptosis inhibitors on tumor survival and subsequent immunotherapy. These results warrant further in deep investigation to measure the effect of various ferroptosis inducing methods in multiple cancer types under different treatment-conditions.

Compared with the tumor-specific CD8^+^ T cells, the tumor-derived Tregs are less frequently subjected to ferroptosis due to the low levels of LPO in TME (40). But the ferroptosis could be specifically induced in Tregs upon the genetic depletion of *GPX4* and subsequently facilitated the T helper 17 (Th17) cell response to promote the antitumor immunity which can be rescued by the treatment of ferroptosis inhibitors. However, the effect of GPX4 inhibitors on tumor immunity and tumor growth through Tregs in different cancer types needs further investigation (41, 42).

#### 3.1.2 Cross talk between ferroptosis and B cells

B cells can be converted into plasma cells to produce antibodies and participate in immune response, its subpopulations include B1, B2 and regulatory B cells. B2 cells are derived from the bone marrow and can be further classified into follicular B (FOB) and marginal zone B (MZB) cells. A recent study showed that the growth of B1 and MZB cells but not follicular B2 cells was regulated by GPX4 due to the higher expression of *CD36* in B1 and marginal zone (MZ) B cells. In line with this notion, depletion of *GPX4* induced lipid peroxidation and ferroptosis in B1 and MZ B cells (43). However, whether the ferroptosis inducers and inhibitors could efficiently trigger the ferroptosis in B cells and affect their function and regulate the B cell-mediated tumor immunity need further investigation.

#### 3.1.3 MDSCs are the ferroptosis resistant cell types in TME

MDSCs are the common cell type in tumors and exhibit immunosuppressive function in the TME. Accumulating evidence suggested that the tumor-infiltrating MDSCs are resistant to ferroptosis-mediated cell death because the system Xc- and the neutral ceramidase N-acylsphingosine amidohydrolase (ASAH2) are highly expressed in MDSCs (44). The results from other studies suggested that the deficiency of the metabolic conversion of arachidonate (AA) to AA-PLs and peroxidation in MDSCs might confer resistance to ferroptosis (45).

#### 3.1.4 Ferroptosis affects the polarization of macrophages

M1 and M2 macrophages are the two polarized phenotypes of activated macrophages. Tumor-associated macrophages (TAMs) in the TME predominantly demonstrated an M2-like phenotype. The induction of ferroptosis on these cells by ferroptosis inducers is thought to be a potentially exciting therapeutic strategy to reverse the immunosuppressive TME. However, the M1 subtypes are resistant to *GPX4* depletion-induced ferroptosis partially because the M1 macrophages generated higher levels of inducible nitric oxide (NO) synthase (iNOS) and NO radical (NO•). The production iNOS and NO• is inhibited in the M2 macrophages (46). Given that M1 macrophages exhibited the antitumor function in TME and the transformation of M2 phenotypes into M1 phenotypes is an important strategy to enhance tumor immunity. The population of M2 TAM was reduced upon the depletion of *GPX4* in TAMs, while the survival of M1 TAMs was not affected (46). Furthermore, the Platelet Membrane-Camouflaged Magnetic Nanoparticles, a pro-ferroptotic nanoparticles, could trigger a tumor-specific immune response and effectively repolarize the M2 phenotype to the M1 phenotype by inducing ferroptosis (47). It was also reported that the M1 and M2 polarization could be stimulated by the alteration of ferritin, ferroportin, and iron levels which are the important iron metabolism-related components (48). The cancer cells with ferroptosis induced by excessive iron overload could release the 8-OHG to promote the TAMs infiltration and M2 polarization through the activation of stimulator of interferon genes protein (STING)-mediated DNA sensor pathway (49). Hence, investigating the regulation function of ferroptosis on macrophages will provide new options for the development of ferroptosis-targeted therapy in cancer treatment.

#### 3.1.5 Relationships between ferroptosis and DCs and NK cells

Dysregulation of the function of NK cells promotes tumorigenesis and cancer development due to their central roles in antitumor immunity (50). It was reported that the levels of proteins which is highly associated with ferroptosis such as lipid peroxidation, and oxidative damage were elevated in tumor-associated NK cells (51). The glucose metabolism of NK cells could be inhibited by lipid peroxidation-associated oxidative stress which leads to the abnormality of NK cells in TME. However, the suppressed glucose metabolism of NK cells can be rescued by the activation of nuclear factor E2-related factor 2 (NRF2) to further recover the antitumor function of NK cells (51, 52). Further investigations should be conducted to explore whether the ferroptosis inducers and inhibitors have a consistent effect on the function of tumor-associated NK cells.

DCs, the antigen-presenting cells, play important roles in T cell-dependent immunity by mediating the naïve T cell activation. The ferroptosis was correlated with tumor-associated DCs because the expression of the elevated lipids which is highly associated with ferroptosis hampered the antigen presenting function of DCs (53, 54). However, direct evidence should be provided in the future to explore whether the ferroptosis inducers and inhibitors could affect the function of tumor-associated DCs in the TME.

### 3.2 Major metabolism processes and ferroptosis in TME

The oxidation of phospholipids, the lack of lipid peroxide repair capability and the overloading of redox-active iron are the three hallmarks of ferroptosis which suggested the biological metabolisms might highly correlated with ferroptosis. Therefore, the metabolism of amino acid, iron, lactate and lipid in TME plays important role in the occurrence and regulation of ferroptosis.

#### 3.2.1 The amino acid metabolism in the TME regulates ferroptosis

Amino acids are important components in the TME and amino acid metabolism is highly associated with ferroptosis. The cysteine, glutamate, and glycine consist of the tripeptide GSH which plays important roles in the GPX4-mediated ferroptosis in cancer cells (55). Consistently, the DPI2 and cisplatin can trigger the ferroptotic cell death by inhibiting GSH synthesis (26). Notably, in pancreatic cells, ferroptosis could be promoted by decreased exogenous cystine due to the inhibition of cytosolic aspartate aminotransaminase (GOT1) (56). Furthermore, ferroptosis could be induced by elevated levels of extracellular glutamate by hampering the system Xc^−^ function and extracellular glutamine is considered an inducer of ferroptotic cell death (36). Furthermore, the effector CD8^+^ T cells could produce IFNγ to reduce the cancer-associated fibroblasts (CAFs) released cysteine and GSH and subsequently decreased the expression of *SLC7A11* and *SLC3A2*. The cystine uptake was inhibited in cancer cells with low expression level of *SLC7A11* and *SLC3A2* which ultimately triggered the tumor lipid peroxidation and ferroptosis (37). Importantly, dysregulation of intracellular GSH by reducing extracellular cystine and cysteine or other methods could trigger ferroptotic cell death in combined with PD-L1 blockade (57). Altogether, amino acid metabolism in TME plays critical roles in ferroptotic cell death and tumor development (58).

#### 3.2.2 The iron metabolism in the TME determines ferroptosis

Iron, an important element in many critical biological processes, plays vital roles in the regulation of ferroptosis through modulating ROS *via* the Fenton reaction. Deficiency in the GSH-mediated anti-oxidative system resulted in increased iron-dependent lipid peroxides which induced ferroptotic cell death. The ferritin protein is highly expressed in M1 macrophage with low expression of ferroportin, however, the M2 macrophages expressed high levels of ferroportin and low levels of ferritin. Therefore, the M2 macrophages preserve the function to promote iron release and disturbance of iron metabolism in cancer cells (59). It was also reported that the M1 and M2 macrophages polarization could be stimulated by the alteration of ferritin, ferroportin, and iron levels which are the important iron metabolism-related components (48). The cancer cells with ferroptosis induced by excessive iron overloading could release the 8-OHG to promote the TAMs infiltration and M2 polarization through the activation of stimulator of interferon genes protein (STING)-mediated DNA sensor pathway (49). The TAMs and neutrophils are important to iron metabolism as the iron providers in TME. One important mechanism is that macrophages can take a plenty of iron from senescent red blood cells, and along with the macrophages, the neutrophils can also produce iron-related proteins Lcn2 and lactoferrin which play critical roles in iron metabolism (60). Furthermore, the other TME components hepcidin, tumor necrosis factor (TNF)-α, IFNγ and interleukin (IL)-6 play important roles in regulating the iron metabolism by affecting the iron-regulatory related genes in TME (61–63). Therefore, the regulation of iron metabolism in TME and cancer cells is of significant importance for the induction of ferroptosis (58).

#### 3.2.3 The metabolites lactate in the TME affects ferroptosis

Under physiological conditions, most organs or tissues utilize the lactate as a fuel source but during tumorigenesis, the lactate is mainly produced by tumor cells and CAFs in the TME. Moreover, it was reported that lactate plays important roles in regulating TME and is highly associated with lipid biosynthesis and oxidative stress resistance as a signaling molecule beyond an energy source (64–66). The generation of some lipid substances such as acetyl-CoA and citrate was regulated by the levels of extracellular lactate (67). Importantly, lactate can be utilized by Treg cells in the TME to inhibit the function of effector T cells (42) and Tumor cell-derived lactate could promote the polarization of TAM into the M2 phenotype (68). Furthermore, it was also reported that the liver cancer cells were resistant to oxidative stress induced ferroptosis through the elevated formation of MUFAs induced by lactate (69). Therefore, these findings demonstrated that lactate might play an essential role in regulating ferroptotic cell death and negatively affects the immune system in TME by providing an immunosuppressive environment (69). However, the detailed mechanisms of lactate in regulating ferroptosis in the tumor through the immune system and whether ferroptosis inducers could trigger ferroptosis through lactate need further investigation.

#### 3.2.4 Lipid metabolism in the TME regulates ferroptosis

Given that lipid peroxidation is one of the hallmarks of ferroptosis and is highly associated with lipid metabolism, lipid metabolism is an important mediator for the occurrence of ferroptosis. Different oxidized lipid metabolites could be released by cancer cells undergoing ferroptosis to influence the function of immune cells. The prostaglandin E2 (PGE2) was reported to change the TME condition by inhibiting the function of NK cells, cytotoxic T cells, and conventional type 1 dendritic cells (cDC1s) (70). The intracellular fatty acid (FA) mainly comes from the blood and lymphatic vessels and the level of FA is precisely regulated by the cell condition and external stimuli. FA oxidation is an important energy resource of cancer cells. The TME can affect the utilization of lipids by cancer cells through interaction with adjacent stroma. In line with this notion, the lipid metabolism can be regulated by the tumor hypoxia environment through the activation of hypoxia-inducible factors (HIFs) (71). However, during tumor development, the tumor cells could release some FAs into the TME to affect the function of infiltrating immune cells such as the transition from TAMs to M2 phenotype (72). Furthermore, the antigen presentation function of DCs was impaired when the lipid accumulated in DCs and further led to the impairment of T cell responses and anti-tumor immunity (53). Therefore, lipid metabolism is an important regulator for ferroptosis in the TME and cancer cells (58).

## 4 Targeting ferroptosis for tumor therapy

Given the suppression function of ferroptosis on tumorigenesis, it will be promising to develop new strategies to trigger ferroptosis in tumor cells to facilitate the elimination of cancer cells. The common strategies in inducing ferroptosis mainly focused on targeting its upstream genes or signaling pathways which regulate ferroptosis markedly.

The ferroptosis inducer erastin was well studied in inhibiting tumor cell growth *via* ferroptosis, and it targets the system Xc^−^ to reduce the glutathione (GSH) level and targets VDAC2/3 to generate reactive oxygen species (ROS) (73, 74). Moreover, Salazosulfapyridine could inhibit system Xc^−^ to trigger ferroptosis and sorafenib was found to inhibit tumor cell growth by triggering ferroptosis (75). In line with these findings, negative regulation of ferroptosis by a nuclear factor, erythroid 2 like 2 (NRF2) (76) and metallothionein (MT-1G) (77) promotes the hepatocellular carcinoma (HCC) cells growth and also contributes to sorafenib resistance. On the contrary, the treatment of piperazine erastin (PE), an analog of erastin, inhibited tumor growth *in vivo* xenograft models by inducing ferroptosis (26). Furthermore, a plenty of small molecules including the RAS Selective Lethal (RSL) small molecules RSL3, RSL5, artesunate (ART), dihydroartemisinin (DHA) and ferroptosis-inducing agents (FINs) were reported to inhibit various cancer cell growth by inducing ferroptotic cell death, tumor cells treated by the ferroptosis inhibitor ferrostatin-1 showed an enhanced ability of cell proliferation and tumor growth (27, 78–81).

Due to the high expression level of the GSH-dependent enzyme GPX4 in various cancers and its function in inhibiting the generation of lipid ROS, targeting GPX4 might be an efficient method to induce ferroptosis in cancer cells (55). In line with this notion, Cisplatin and DPI2 can induce ferroptotic cell death by inhibiting GSH synthesis (26). RSL could induce ferroptosis by inactivating GPX4 in the VDAC2/3 and protein synthesis dependent manner. The potent ferroptosis inducer FIN56 could trigger ferroptosis by facilitating the degradation of GPX4 or inducing squalene synthase (26). Furthermore, the other GPX4 inhibition-based ferroptosis inducers FINO2 and withaferin A could inhibit GPX4 by oxidizing Fe and elevating the Fe^2+^ level, respectively (82, 83). Notably, Superfluous Fe ions were found to trigger cell death by inducing ferroptosis (84). It was reported that the pancreatic ductal adenocarcinoma cells are sensitive to the ferroptosis induced by artemisinin but less sensitive to apoptosis- mediated cell death (48). Importantly, the bromodomain proteins inhibitor JQ1 could inhibit tumor cell growth by inducing ferroptosis *via* decreasing the activity of histone methylase G9a or enhancing the histone deacetylase SIRT1 activity (85). Recently, a study tested the anti-tumor effect of the newly developed therapeutic strategy for ferroptosis-based therapy combined with iron nanoparticles and cancer-specific gene interference by sgRNAs in variant cancer cells (86). The cancer cells with depletion of two iron metabolic genes (*FPN* and *LCN2*) by Cas13a were treated by iron nanoparticles at the same time and the cells exhibited significant growth suppression due to the enhanced ferroptosis. The result was further confirmed by the *in vivo* mice experiments using adeno-associated virus delivering system to introduce iron nanoparticles and genes into mice (86).

**Figure 3:**
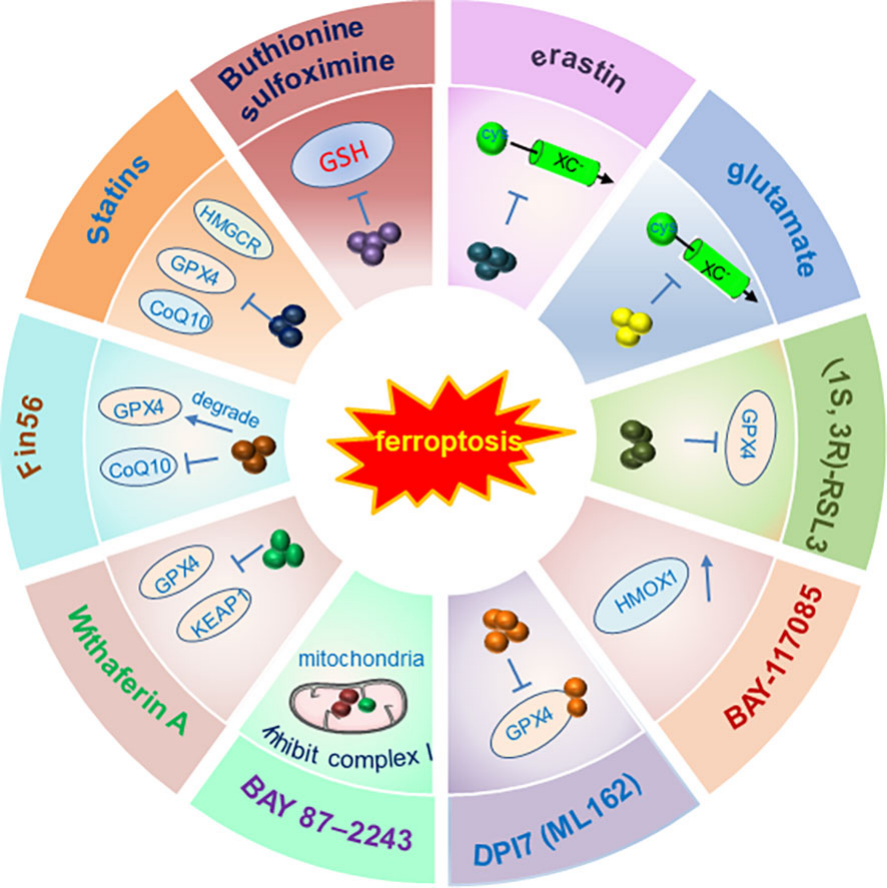
Schematic illustration of ferroptosis inducers targeting Xc^-^/GPX4 pathway. The developed ferroptosis inducers and their functional mechanisms were illustrated in the figure. And these ferroptosis inducers are designed mainly to target the Xc^-^/GPX4 pathway. Not all the currently reported small molecules were listed in the figure and only one of the ferroptosis inducers was illustrated in the figure as a representative of each kind of inducers. Abbreviations: FIN, ferroptosis-inducing compound; GPX4, glutathione peroxidase 4; GSH, glutathione; HMGCR, 3-hydroxy-3-methylglutaryl-CoA reductase; HMOX1, heme oxygenase-1; KEAP1, kelch-like ECH-associated protein 1. Modified according to (48).

**Figure 4:**
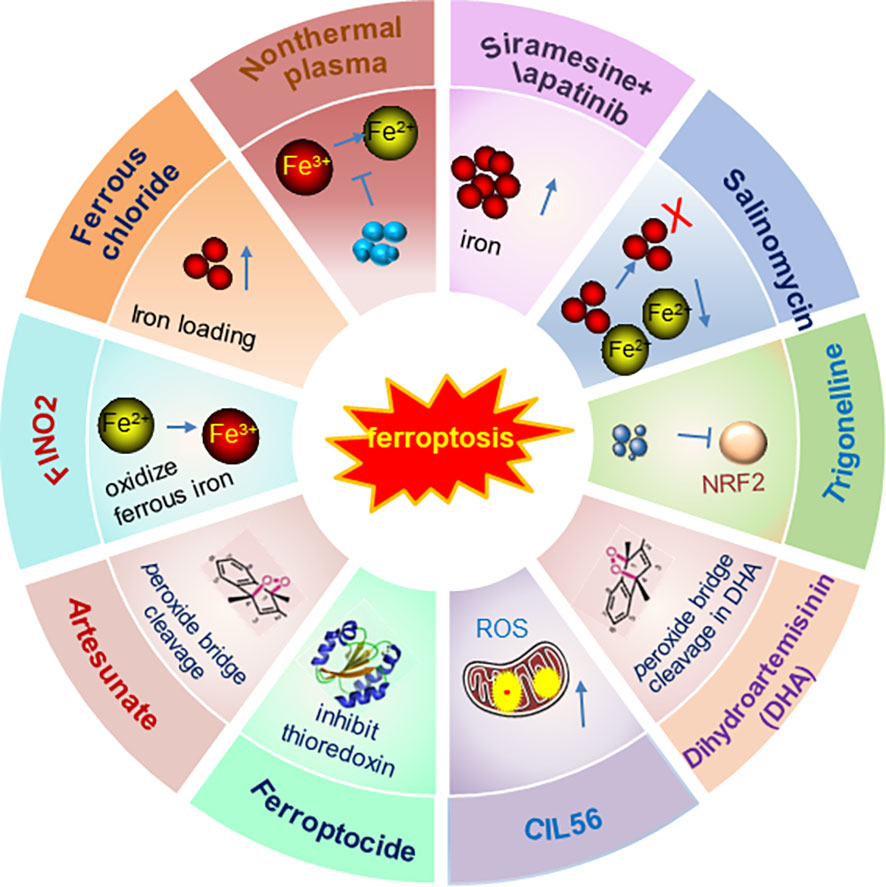
Schematic illustration of ferroptosis inducers targeting iron overloading. The developed ferroptosis inducers and their functional mechanisms were illustrated in the figure. And these ferroptosis inducers are designed mainly to target the iron overloading pathway. Not all the currently reported small molecules were listed in the figure and only one of the ferroptosis inducers was illustrated in the figure as a representative of each kind of inducers. Modified according to (48).

**Figure 5:**
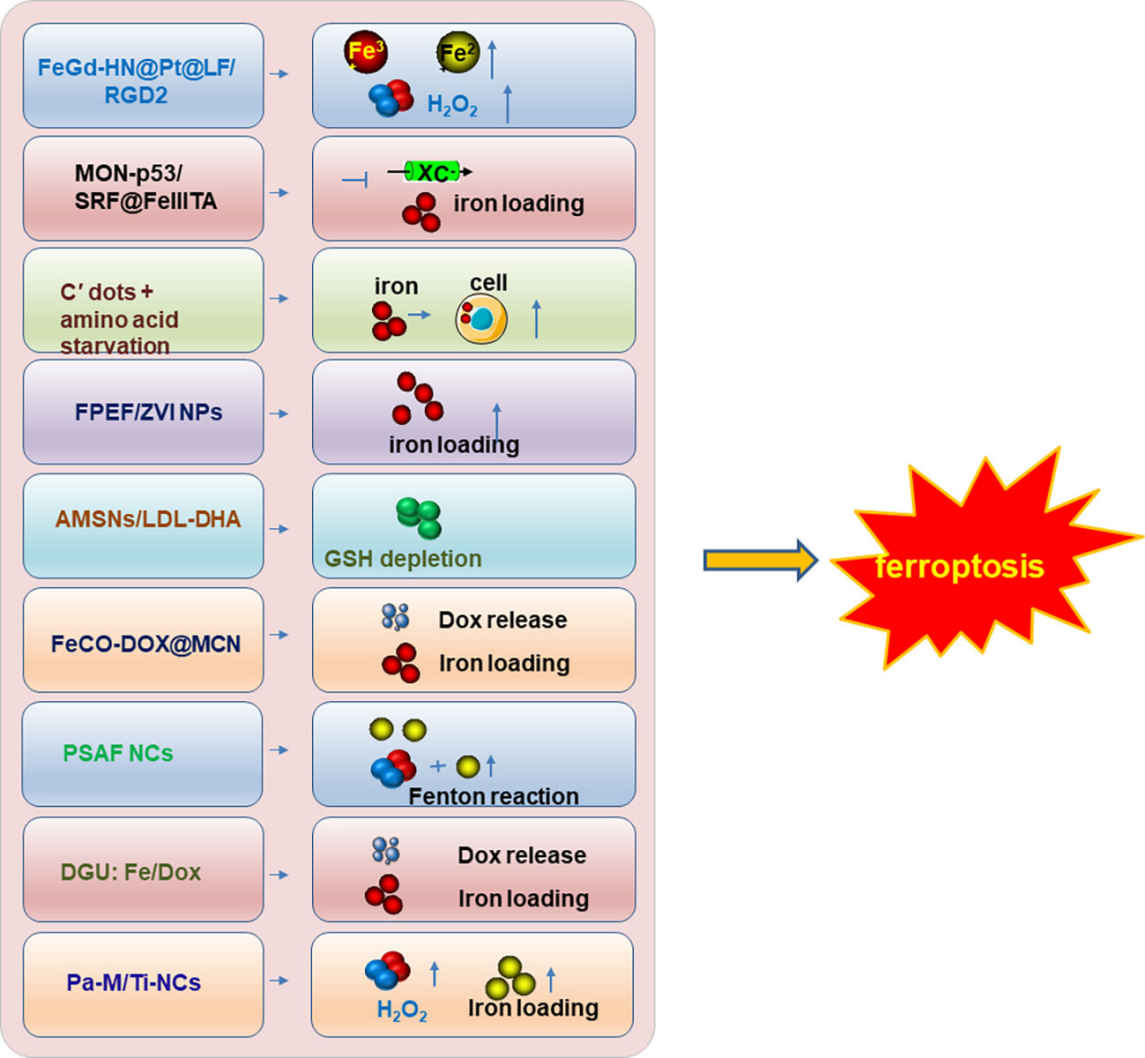
The nanoparticle inducers to trigger ferroptosis. The developed nanoparticle inducers to trigger ferroptosis and their functional mechanisms were illustrated in the figure. And these ferroptosis inducers are designed based on the nanoparticles. Not all the currently reported nanoparticle inducers were listed in the figure and only one of the ferroptosis inducers was illustrated in the figure as a representative of each kind of inducers. Modified according to (48).

Therefore, inducing ferroptosis by small molecules (**Figures 3**, **4**) and nanoparticles (**Figure 5**) to inhibit system Xc^−^ and prevent cystine import, inhibit cystine uptake, directly or covalently inhibit GPX4, to induce the degradation or depletion of GPX4, will provide a novel opportunity for the treatment of various cancers (48).

## 5 Potential roles of ferroptosis in drug resistance of tumor therapy

Importantly, the occurrence of resistance to drugs or currently used oncological therapies hampered the wide application of therapeutic strategies and is a huge challenge for cancer treatment in the preclinical or clinical stage. It is urgent to explore potential approaches to overcome drug resistance in various cancers.

Intriguingly, ferroptosis has been reported to play important roles in helping to reverse drug resistance (87). Elevated expression of *Frizzled 7 (FZD7)* was correlated with platinum resistance in ovarian cancer cells partially due to the decreased ferroptosis regulated by the *FZD7*/*Tp63/GPX4* signaling axis (88). Consistently, the metastatic colorectal cancer cells with KRAS mutation which is resistant to the single treatment of cetuximab are sensitive to the combined treatment of cetuximab and β-elemene, a novel natural ferroptosis inducer (89). The poly (ADP-ribose) polymerase (PARP) inhibitor olaparib showed limited treatment effect on BRCA-proficient patients but the combination of FINs and olaparib could efficiently induce the sensitivity of BRCA-proficient ovarian cancer cells to olaparib in both cells and xenograft models (90). Although the clinical trials show that a wide range of tumor types benefit from Gefitinib, it exhibited a limited therapeutic effect on TNBC. Notably, the combination treatment of GPX4 inhibition and gefitinib could enhance the sensitivity of TNBC cells to gefitinib treatment (91). Furthermore, lung cancers with EGFR mutation which acquired resistance to EGFR tyrosine kinase inhibitors (EGFR-TKIs) are found to be sensitive to ferroptosis mediated cell death. And the histone deacetylase inhibitor vorinostat was reported to increase the sensitivity to EGFR-TKIs by inducing ferroptosis through reducing the expression of SLC7A11 (xCT) in lung cancer cells (92). Moreover, it was reported that SOX2 confers resistance to ferroptosis through upregulation of SLC7A11 expression in lung cancer stem-like cells (CSLC) which implies that SOX2 could be a potential therapeutic target for cancer treatment (93). Notably, sunitinib, a tyrosine kinase inhibitor achieved promising anti-tumor effects on patients with renal cell carcinoma and artesunate treatment indicated a significant inhibition effect on the sunitinib-resistant renal cell carcinoma cells by inducing ferroptosis through suppressing GPX4 (94). Taken together, these findings suggested that the combined therapy of ferroptosis induction and other treatment strategies would be promising therapeutics to overcome drug resistance during cancer treatment (95).

## 6 Ferroptosis and tumor immunity

In recent years, tumor immunotherapy such as the reactivation of the function of immune cells and the induction of immune responses to tumor cells by CAR-T cells, ICIs, cytokine therapies and dendritic cell vaccines achieved promising progress in the treatment of multiple cancer types. However, there are still a huge number of cancer patients less sensitive to immunotherapy, which warrants further in deep investigation to explore the underlying mechanisms and enhance the therapeutic effect or reverse the insensitive states of “cold” tumors. Like other forms of PCD that showed variant correlation with tumor immunity, the potential roles of ferroptosis in regulating tumor immunity attracted enormous attention from the day it was identified in 2012. Accumulating evidence has indicated that ferroptosis plays critical roles in the regulation of tumor immunity, implying that targeting ferroptosis might be a potential method to enhance the efficacy of immunotherapy.

Ferroptosis was considered immunogenic cell death (ICD) because the cells undergoing ferroptosis usually release damage-associated molecular patterns (DAMPs) or lipid metabolites, which could modulate the cellular immune response by affecting the macrophage-mediated phagocytosis and immune cell function. The ferroptosis-induced dendritic cell maturation *in vitro* also contributes to its characteristics as ICD (34, 96). A study firstly demonstrated the immunogenicity of ferroptosis in preclinical models which is highly correlated and modulated the immune system in a time-dependent manner (34). Briefly, RSL3 treatment induced ferroptosis by inhibiting GPX4 in mouse fibrosarcoma MCA205 cells. Subsequently, the effect of ferroptotic MCA205 cells on the phenotypic maturation of mouse bone-marrow-derived dendritic cells (BMDCs) was investigated in their co-culture system. Their results demonstrated that the early ferroptotic cancer cells (treatment of RSL3 for 1 h) but not the late ferroptotic cancer cells (treatment of RSL3 for 24 h) promoted the maturation of BMDCs, exhibiting the capability of BMDCs to eliminate the late ferroptotic cancer cells through phagocytosis. In line with this finding, the early or late ferroptotic MCA205 cells were subcutaneously injected into the flank of immunocompetent C57BL/6 J mice and immunocompromised *Rag2* ^−/−^ mice. Only the early ferroptotic MCA205 cells could trigger a vaccination-like effect to eliminate cancer cells by triggering the protective immune response in C57BL/6 J mice but not immunocompromised *Rag2* ^−/−^ mice. Notably, the immunogenicity of ferroptotic cells was mediated by the release of extracellular high-mobility group box 1 (HMGB1) and adenosine triphosphate (ATP). Consistently, blockage of the ATP receptor P2X7 by the treatment of oxiATP damaged the vaccination-like effect induced by early ferroptotic cancer cells (34, 97). Moreover, it was reported that the HMGB1 released by cancer cells undergoing ferroptosis could increase the tumor necrosis factor α (TNFα) of macrophages (98). Notably, photodynamic therapy (PDT) or Fenton reaction-induced ferroptosis promotes the susceptibility of calreticulin (CRT) of tumor cells and subsequently promotes the dendritic cell maturation and cytotoxic T lymphocyte (CTL) infiltration (99). Interestingly, the neoantigens can be released by the cancer cells upon ferroptosis. For example, the pancreatic cancer cells undergoing ferroptosis could release the mutant KRAS^G12D^ proteins (neoantigens) to promote the differentiation of tumor-associated macrophages to the M2 phenotype (100). Whether the ICD could also be triggered by ferroptosis warrants further in deep study and will provide more evidences to illustrate the function of ferroptosis.

Besides cancer cells, ferroptosis could also be induced in the immune cells in certain physiopathological conditions and it will subsequently trigger various immune responses. It is reported that T cells with GPX4 deficiency undergo ferroptosis and negatively affect their anti-virus or anti-infection function (101), and the ferroptosis is inhibited in GPX4 or FSP1 overexpressed T cells without affecting their effector function. However, depletion of ACSL4 in CD8^+^ T cells could inhibit the ferroptosis accompanied by impaired immune functions (40). The antitumor immunity of immune cells will be impaired upon the accumulation of fatty acids (FAs) in the TME due to induced ferroptosis. However, the deficiency of CD36, a scavenger receptor, could reactivate the antitumor function by inhibiting the ferroptosis of T cells (102). Moreover, GPX4 is essential for the normal function of B1 and marginal zone (MZ) B cells due to the high expression of CD36 on the cells which increased the occurrence of ferroptosis (43). Interestingly, the function of M1 and M2 macrophages can be affected by ferroptosis but the M1 macrophages are less sensitive to ferroptosis compared to M2 due to the reduced lipid peroxidation in M1 macrophages (46).

However, the notion of ferroptosis as an ICD was challenged by the finding (103). The study verified the immunogenicity of ferroptotic cancer cells by measuring several parameters such as the efficacy to trigger vaccination-like effect, the release of DAMP, chemokines, IFN and cytokines, and the correlation with dendritic cells (DCs) transcriptional regulation and antigen presentation. The authors generated an inducible ferroptotic model and classified three stages of ferroptosis including initial, intermediate and terminal ferroptosis. The characteristics of the three stages are lipid peroxidation, ATP release, and HMGB1 release and loss of plasma membrane integrity. The study using the co-culturing system for ferroptotic cancer cells and DCs indicated that the antigen-presenting cells such as DCs and adaptive immune response were negatively affected by the ferroptosis. This provided a new challenge in the development of antitumor strategy by inducing ferroptosis in cancer cells (103). The above conflicting findings might be due to the capability to release the DAMP and cytokines in the former study (34). However, there might be some other reasons for the controversy. Early ferroptosis triggered by the short time treatment of ferroptosis inducers such as RSL3 was not a complete cell death because the chemical ferroptosis inducers could not fully kill the cancer cells, after they were removed, some living cancer cells still existed in the treated cells which was confirmed by the two groups (34, 103). The latest study avoided the possibility of the existence of living cells, and it inhibits GPX4 by doxycycline to induce a synchronized and complete ferroptosis (34).

Given the complicated relationship between ferroptosis and immunity, a full understanding of the function of ferroptosis and the development of novel therapeutic strategies based on ferroptosis will shed light on the improvement of tumor immunotherapy.

## 7 Ferroptosis and tumor immunotherapy

Tumor immunotherapy, especially the ICIs, has achieved great progress in treating multiple cancer malignancies in recent years. However, there are still a large number of cancer patients who showed fair response or no response to immune checkpoint inhibition due to various mechanisms. Therefore, how to improve the treatment efficacy of immunotherapy attracted numerous attentions in the cancer therapeutic research field. Notably, the PCD such as ferroptosis was found to play essential roles in antitumor immunity and in regulating the immune response to ICIs. Given that ferroptosis is highly correlated with tumor immunity and TME regulation, ferroptosis-mediated cell death might be an important factor to affect the immune cell response to ICIs and the therapeutic efficacy of ICIs. The sensitivity to ferroptosis might be a cancer type-dependent and affected by cancer types and the immune state of patients.

In line with this notion, ferroptosis was found to play critical roles in anti-tumor immunity of T cells and in modulating the efficacy of tumor immunotherapy (16). It was reported that the degradation of cysteine in cancer cells induced by cyst(e)inase treatment leads to ferroptotic cell death because of the reduction of intracellular GSH and elevation of ROS (104). A recent study tested the interaction of ferroptosis and immunotherapy in cancer cells and found that ferroptosis could enhance the anti-tumor efficacy of immunotherapy *via* the elevated ferroptosis-specific lipid peroxidation and reduced cystine uptake induced by immunotherapy-activated cytotoxic CD8^+^ T cells (57). Specifically, CD8^+^ T cells activated by ICIs (e. g., anti-PD-L1 antibodies) reduce the expression of *SLC3A2* and *SLC7A11* by releasing the interferon gamma (IFNγ) to activate the JAK-STAT1 pathway. As a result, the lipid peroxidation was elevated and ferroptosis was induced in cancer cells (57). This finding was further verified in a cyst(e)inase mediated depletion of cystine or cysteine mice models, the ICIs treatment indicated a synergistic effect on tumor cells growth inhibition by inducing ferroptosis and enhancing T cell-mediated anti-tumour immunity. Furthermore, the anti-tumor efficacy of nivolumab in melanoma patients was negatively correlated with the expression of *SLC3A2*, and was positively correlated with IFNγ and CD8 (57). This study provided detailed evidence for the ferroptotic cell death induced by T cells and suggested a promising therapeutic approach to enhance immunotherapeutic efficacy by targeting this pathway in combination with immunotherapy in cancers.

It was well known that ferroptotic cells could regulate the innate immune system and the inflammation response *via* the released multiple damage-associated molecular pattern molecules (DAMPs). Cancer and non-cancer cells with ferroptosis induced by the treatment of erastin, sorafenib, RSL3, and FIN56 could release the high mobility group box 1 (HMGB1), one of the DAMPs. Furthermore, the promotion of inflammation in macrophages by ferroptotic cancer cells released HMGB1 depended on the advanced glycosylation end-product specific receptor (AGER) (98). Therefore, HMGB1-AGER pathway plays an important role in modulating ferroptosis-mediated inflammatory response (105). Targeting HMGB1 might achieve some potential therapeutic effects in cancers *via* the regulation of ferroptosis. In line with this notion, in an *in vivo* study using the orthotopic, syngeneic mouse models of basal-like breast or non-small cell lung cancer, targeting extracellular HMGB1 by various inhibitors remodeled the immune microenvironment by dramatically reducing the regulatory T lymphocytes and monocytic/granulocytic MDSC, increasing the M1/M2 macrophages, activating DC and pDC. Therefore, HMGB1 blockage significantly inhibited tumor growth and enhanced the immunotherapeutic efficacy of anti-PD-1/PD-L1 treatment (106). Given that the ASAH2 plays an important role in positively regulating the MDSC, and ASAH2 was found highly expressed in tumor-infiltrating MDSCs in colon carcinoma (44). NC06, an ASAH2 inhibitor, could induce ferroptosis in MDSC by promoting the protein stability of p53 and the expression of *Hmox1*. Therefore, targeting ASAH2 to trigger ferroptosis in MDSC to enhance the CD8^+^ T cell activity might facilitate cancer immunotherapy (44). BEBT-908, the PI3K and HDAC inhibitor, could enhance the efficacy of anti-PD1 therapy and inhibit tumor cell growth by triggering immunogenic ferroptosis. Mechanistically, BEBT-908 treatment resulted in hyperacetylation of p53, promoted the expression of MHC class I and activation of endogenous STAT1/IFNγ signaling (107).

Improved efficacy of immunotherapy in combination with radiotherapy was found during the treatment of cancer patients but the underlying mechanisms are not fully investigated (108). It was reported that radiation could promote lipid synthesis and lipid peroxidation which are the hallmarks of ferroptosis, by inducing ROS production and increasing *ACSL4* expression (109–111). Furthermore, radiotherapy in combined with immunotherapy significantly induced ferroptosis by decreasing cystine uptake due to the DNA damage-activated kinase ATM and IFNγ-mediated downregulation of SLC7A11 (14, 57). Furthermore, unexpected resistance to immunotherapy such as PD-L1 blockade is also frequently observed during immunotherapy in cancer patients. Ferroptosis might contribute to immunotherapeutic resistance. The expression of *SLC3A2* is upregulated by TYRO3 signaling pathway which is highly expressed in anti-PD-1 resistant tumors. Notably, in a TNBC syngeneic mouse model, anti-PD-1 therapy in combination with TYRO3 receptor tyrosine kinase (RTK) inhibitor could enhance the sensitivity of anti-PD-1 resistant tumors to anti-PD-1 therapy by inducing the ferroptosis (16, 112). The insufficient immunogenicity of the tumor cells is another reason for the poor or low response to current cancer immunotherapy. Therefore, eliciting immunogenicity in some tumors by inducing ferroptosis might be helpful to enhance the immune response. In a study, an intracellular-acidity-activatable dynamic nanoparticle containing glutathione peroxidase 4 inhibitor RSL-3 was designed. The effect of the nanoparticle to initiate antitumor immune responses and to induce the secretion of IFN-γ by cytotoxic T lymphocytes *via* inducing the ferroptotic cell death in B16-F10 melanoma tumor and 4T1 breast tumors was evaluated (113). Interestingly, the nanoparticle in combination with the blockade of programmed death ligand 1 significantly increased the tumor infiltration of cytotoxic T lymphocytes and efficiently retarded the tumor growth and lung metastasis (113). Altogether, targeting ferroptosis in combination with immunotherapy might be a promising therapeutic strategy to enhance their anti-tumor effect.

## 8 Conclusion and outlook

Taken together, ferroptosis play important roles in regulating cancer development, treatment response, drug resistance and the efficacy of immunotherapy. Therefore, targeting ferroptosis alone or in combination with immunotherapeutic strategies such as ICIs, might be a promising therapeutic strategy to enhance the efficacy of tumor immunotherapy and efficiently inhibit tumor growth.

Currently, most evidence supports the notion that ferroptosis induction is the potential strategy to suppress cancer development. Accordingly, the discovery and development of various strategies to induce ferroptosis attracted enormous attention and have achieved great progress (**Figures 3**-**5**). Therefore, a plenty of mall molecules (**Figures 3**, **4**) and nanoparticles (**Figure 5**) were tested in inducing ferroptosis to inhibit cancer development (48). It should also be kept in mind that ferroptosis has dual effects: ferroptosis can be not only a tumor suppressor, but also an initiator of cancers and other diseases. Ferroptosis can be induced in immune cells in TME and hampers the anti-tumor function of immune cells. Furthermore, it is reported that ferroptosis is highly associated with the onset of various human diseases including cardiovascular diseases (myocardial infarction, reperfusion damage, and heart failure). Importantly, inhibition of cardiac ferroptosis but not cardiac ferroptosis induction is considered an effective therapeutic strategy for cardiovascular disorders (114). However, compared with the great progress of ferroptosis inducers (**Figures 3**-**5**), the discovery and development of various ferroptosis inhibitors need more focus and warrant further in deep investigations.

Moreover, to avoid the possible adverse effects caused by ferroptosis induction, more attention should be paid to the specificity, treatment timing and dosage of ferroptosis inducers used in cancer therapy. Furthermore, the sensitivity to ferroptosis is another issue which needs to pay attention. It was found that the metabolic plasticity and heterogeneity affect ferroptosis sensitivity (87). Other exogenous components involved in ferroptosis such as iron, selenium, oxygen, cysteine, glutathione, PUFAs, etc. also play important roles in regulating the sensitivity to ferroptosis of the targeting cells. Furthermore, the specific characteristics and biomarkers of ferroptosis which used to determine the induced ferroptosis may vary from the different triggering methods and are important for the development of ferroptosis based therapy. Therefore, the exploration of the specific hallmark of ferroptosis warrants further investigation. Importantly, it was also possible that some agents could induce more than one type of PCD and it will be valuable for the development of PCD based therapy to distinguish the difference and the phenomenon of various cell death (48).

Notably, ferroptosis also play critical roles in overcoming resistance to immunotherapy, traditional chemotherapy and targeted therapy. Therefore, targeting ferroptosis is a promising novel therapeutic strategy as it enhances the efficacy of immunotherapy to overcome drug resistance in cancer. However, although the critical roles of ferroptosis in regulating cancer development, tumor immunity, TME and drug resistance were studied in various preclinical studies, there are still a lot of considerations that should be taken before the practical application of ferroptosis induction. Based on these problems, to precisely develop a therapeutic method targeting ferroptosis is extremely complicated. Furthermore, the ferroptotic process of cancer cells is regulated by the components in TME which still needs to be clearly clarified when we develop the ferroptosis based therapeutics (58). Taken together, a deep understanding of the crosstalk between ferroptosis and tumor cells, immune cells, TME, etc. and the ferroptosis-modulating regulators will broaden and deepen our knowledge of ferroptotic cell death, and shed light on the development of ferroptosis-based therapies, also provide new opportunities to enhance the immunotherapeutic efficacy and overcome the drug resistance (16).

## Author contributions

XG wrote the manuscript with partial help from YL, XZ, Y-GY and XD edited and revised the manuscript. All authors contributed to the article and approved the submitted version.

## Glossary

**Table:**

**Abbreviations**	**Stands for**
CAR-T	chimeric antigen receptor T
ICI	immune checkpoint inhibition
PD-L1	programmed cell death-ligand 1
CTLA-4	cytotoxic T lymphocyte-associated antigen-4
PCD	programmed cell death
GPX4	glutathione peroxidase 4
FSP1	ferroptosis suppressor protein 1
TNBC	triple-negative breast cancer
TME	tumor microenvironment
HNC	head and neck cancer
DFO	desferrioxamine
LSH	lymphoid-specific helicase
CDO1	cysteine dioxygenase 1
TEAD4	TEA domain transcription factor 4
YAP	Yes-associated protein
GSH	glutathione
ROS	reactive oxygen species
NRF2	erythroid 2 like 2
MT-1G	metallothionein
HCC	Hepatocellular carcinoma
RSL	RAS Selective Lethal
ART	artesunate
DHA	dihydroartemisinin
FINs	ferroptosis-inducing agents
PARP	poly (ADP-ribose) polymerase
DAMPs	damage-associated molecular patterns
ICD	immunogenic cell death
GPX4	glutathione peroxidase 4
BMDCs	bone-marrow-derived dendritic cells
HMGB1	high-mobility group box 1
ATP	adenosine triphosphate
TNFα	tumor necrosis factor α
PDT	photodynamic therapy
CRT	calreticulin
CTL	cytotoxic T lymphocyte
DC	dendritic cells
CAFs	cancer-associated fibroblasts
MDSCs	myeloid-derived suppressor cells
LPOs	lipid peroxides
ASAH2	N-acylsphingosine amidohydrolase
AA	arachidonate
NO	nitric oxide
iNOS	inducible nitric oxide (NO) synthase
